# Mixed mating system and variable mating patterns in tropical woody bamboos

**DOI:** 10.1186/s12870-019-2024-3

**Published:** 2019-10-11

**Authors:** Ning Xie, Ling-Na Chen, Yu-Ran Dong, Han-Qi Yang

**Affiliations:** 1 Research Institute of Resources Insects, Chinese Academy of Forestry, Bailongsi, Panlong District, Kunming, 650233 China; 2 Lushan Botanical Garden, Jiangxi Province and Chinese Academy of Sciences, Guling, Lushan District, Jiujiang, 332900 China

**Keywords:** *Dendrocalamus*, Mating system, Paternity analysis, Pollen dispersal, Woody bamboo

## Abstract

**Background:**

So far, little is known in detail about mating systems of woody bamboos. Paternity analysis of offspring improved our understanding of these systems, and contributed to their germplasm conservation and genetic improvement.

**Results:**

In this study, a paternity analysis of offspring from two consecutive mass or sporadically flowering events of *Dendrocalamus membranaceus* and *D. sinicus* were conducted to determine their mating system and pollen dispersal using the program COLONY based on simple sequence repeat (SSR) markers. Two sporadically flowering populations of *D. sinicus* (C1, C2) obtained relatively high paternity assignments rates (69.0–71.4%). Meanwhile, among three populations of *D. membranaceus*, the sporadically flowering population A also had much higher paternity assignments rates (56.4%) than mass flowering populations B1(28.6%) and B2 (42.5%). Both *D. membranaceus* and *D. sinicus* had mixed mating systems while their mating patterns were variable depending on pollination conditions. The maximum pollen dispersal distances were 90 m and 4378 m for *D. membranaceus* and *D. sinicus* populations, respectively, and the mating distances of these two species focused on ranges of ca. 0–50 m and 0–1500 m, respectively.

**Conclusions:**

These results revealed for the first time variable mating patterns in woody bamboos. This suggests half-sib seeds from the same bamboo clump may have different male parents and it is crucial to clarify genetic origin in woody bamboos’ breeding programs. The results also indicate the importance of pollinators in the mating systems of tropical woody bamboos.

## Background

Mating systems are generally defined as the mating patterns among individuals, mainly referring to selfing or outcrossing rates and gene flow [1–4]. Mating systems are the crucial part of plant breeding systems that control genetic exchange and play a key role in determining the genetic structure of plant populations, and have a profound influence on survival and evolution of plant species [2, 4, 5]. Meanwhile, background knowledge of mating systems helps us to develop genetic improvement strategies and establish appropriate conservation measures [6].

Woody bamboos (Poaceae: Bambusoideae) comprise ca. Eighty genera including more than 1200 species, which are mainly distributed in the subtropical and tropical regions of Asia, America and Africa [7, 8]. Woody bamboos are shrub forest species which have important economic value such as food, construction and pulp materials, as well as important roles in water and soil conservation [9, 10]. One of the most compelling biological properties of woody bamboos concerns their unusual flowering phenology [11–14]. There are two common unofficial terms related to flowering and mating systems of bamboos. Firstly, based on origin of woody bamboo forests, bamboo species can generally be divided into wild and cultivated species [12]. Secondly, in terms of the size of flowering forests or populations, woody bamboo flowering events can be divided on the whole into two types, namely mass and sporadic flowering [11, 15, 16]. Due to outstandingly long vegetative growth periods (ca. 20–150 years) among seed plant and uncertainty of flowering events [11], so far, little is known about the mating systems of most bamboo species [7, 10, 15], which greatly hinders studies on their germplasm conservation and genetic improvement [9].

As for characteristics of mating systems, most of the documented woody bamboos were dichogamous and protogynous, and were generally considered to be wind-pollinated species [11, 16–18]. However, it is still in controversy about whether bamboos are obligate outcrossers [16]. Within tropical bamboos, *Dendrocalamus* spp. demonstrated a breeding system suitable for hybridization [16–18]. Recently, paternity analyses of several wild temperate woody bamboo species, such as *Sasa cernua* (sporadic-flowering, [19]), *S. veitchii* var. *hirsuta* (mass-flowering, [20]) and *Phyllostachys edulis* (sporadic-flowering, [21]) were reported. All of those studies were based on results from single bamboo flowering events, and it seemed that studied species were self-compatible. Meanwhile, it appeared that selfing was predominant in sporadic flowering events while outcrossing was predominant in mass flowering events.

**Fig. 1:**
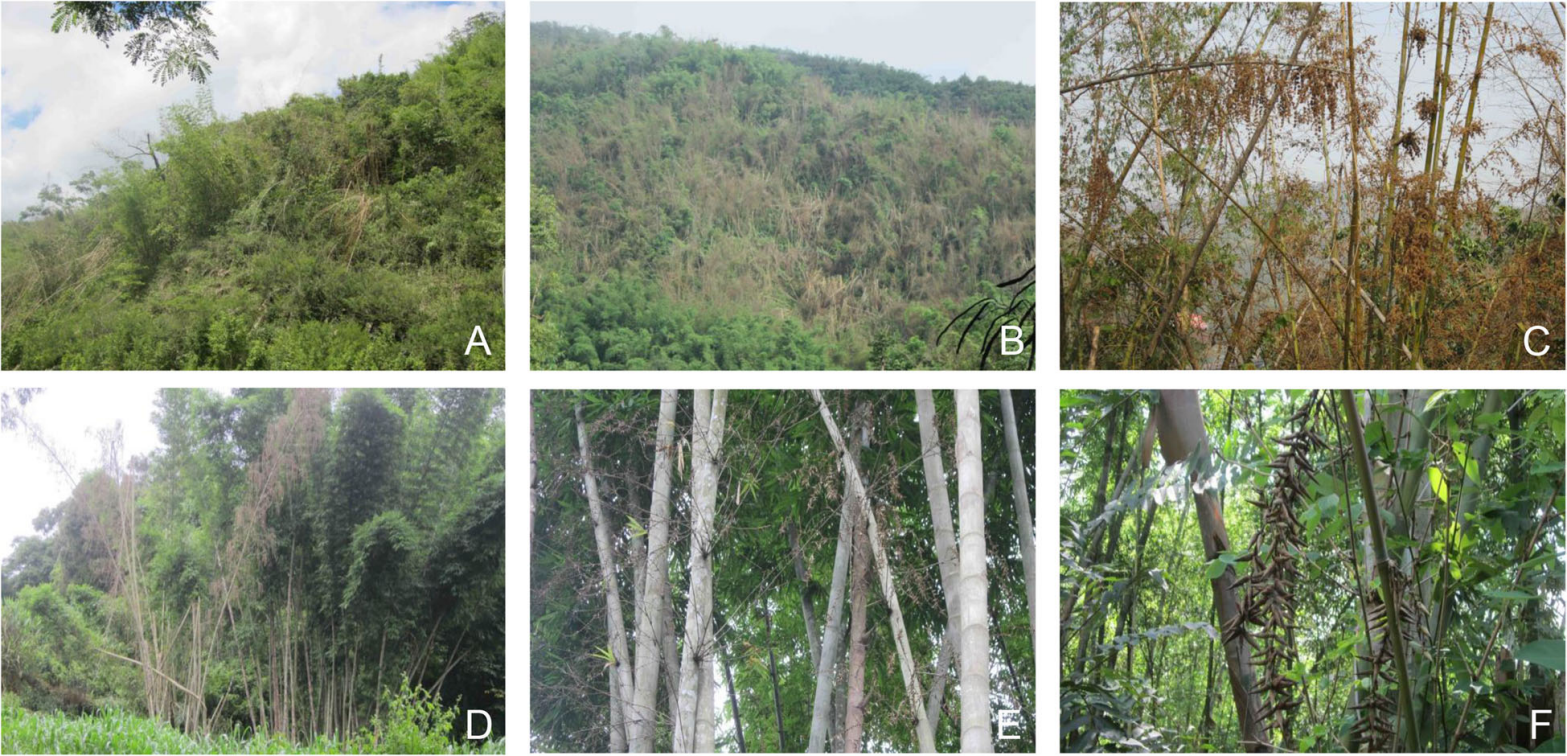
Sporadically flowering (photo **a**, site A), mass flowering (photo **b**, site B) populations and flowering clumps (photo **c**) of *D. membranaceus,* and sporadically flowering population of *D. sinicus* (photos **d–f**, site C) in southwestern Yunnan, China

As one of the regions with the most woody bamboo species in the world, Yunnan Province of China possesses more than 230 native species from 28 genera [10]. From 2008 to 2015, many bamboos experienced mass and sporadically flowering following a prolonged severe drought in Yunnan [22, 23]. In particular, the mass and sporadic flowering of *Dendrocalamus membranaceus* and sporadic flowering of *D. sinicus* were often observed [16, 22, 23] (Fig. 1). This provided a good opportunity for us to investigate the characteristics of bamboo mating systems.

*Dendrocalamus membranaceus* is mainly distributed in middle and lower Lancang-Mekong River valley including Myanmar, Laos, Thailand and China’s Yunnan Province, with an elevation range of 500–1000 m [24]. In Yunnan, *D. membranaceus* often grows into large-scale natural forests, totally covering a region of ca. 30,000 hm^2^ in 2008, and is the largest natural bamboo forest in China [24]. This species is economically important as vegetable crops and raw materials for furniture, construction and industrial paper pulp [7]. *Dendrocalamus sinicus* is endemic to southwestern Yunnan at elevations of 500–1800 m, and is famous for its strongest culms among woody bamboos in the world so that it is an important bamboo species for producing timber [10]. *Dendrocalamus sinicus* is cultivated by local Wa or Dai peoples in Yunnan.

In our previous study about pollination and breeding systems in wild *D. membranaceus* populations and cultivated *D. sinicus* population based on morphology observation and hand pollination experiment, we inferred that both were self-compatible and predominantly outcrossing [16]. Here, we investigated the mating systems of mass or sporadic flowering populations of these two typical tropical woody bamboos, i.e., wild *D. membranaceus* and cultivated *D. sinicus*, based on data from two continuous flowering events and using simple sequence repeat (SSR) molecular markers. We carried out paternity analyses using program COLONY [25], and estimated selfing and outcrossing rates, male reproductive fitness, and pollen dispersal in mass and sporadic flowering populations of *D. membranaceus* and sporadic flowering populations of *D. sinicus*. Our goals were to understand the characteristics of the mating systems of tropical woody bamboos so as to provide a scientific foundation for germplasm conservation and further genetic improvement.

## Results

### Mating system

Outcrossing rates of *Dendrocalamus membranaceus* and *D. sinicus* were estimated using MLTR [5], and the result indicated that both *D. membranaceus* and *D. sinicus* were predominant outcrossing (Table 1). Multilocus outcrossing rate of mass flowering populations (B1, B2) of *D. membranaceus* were 1.000, which was higher than the sporadically flowering population A (t_m_ = 0.643). The single-locus outcrossing rate (t_s_) of population A was highest (t_s_ = 0.843), and population B1 was lowest (t_s_ = 0.001). On the other hand, biparental inbreeding of population B1(t_m_*–*t_s_ = 0.999) was highest among three *D. membranaceus *populations, indicating that individuals within population B1 were close relatives. Meanwhile, population A had the lowest biparental inbreeding (t_m_-t_s_ = *−* 0.200), indicating an excess of heterozygotes.

**Table 1:** Estimation of outcrossing rates of *D. membranaceus* and *D. sinicus* based on MLTR

Species	Population	Multilocus outcrossing rate (t_m_)	Single-locus outcrossing rate (t_s_)	Biparental inbreeding (t_m_-t_s_)
*D. membranaceus*	A	0.643	0.843	– 0.200
	B1	1.000	0.001	0.999
	B2	1.000	0.804	0.196
*D. sinicus*	C1	1.200	1.158	0.042
	C2	0.697	0.724	– 0.027

Outcrossing rates of sporadically flowering populations (C1, C2) of D. sinicus were relatively high, and both multilocus and single-locus outcrossing rates of population C1 were higher than population C2. The values of biparental inbreeding were very low in populations C1 and C2, which were 0.042 and *–* 0.027, respectively. This suggests an excess of heterozygotes within sporadically flowering populations of *D. sinicus*.

### Paternity assignments

Paternity assignments (with 95% confidence) of *Dendrocalamus membranaceus* and* D. sinicus* based on COLONY [25] are given in Table 2, Additional file 1: Table S1 and Additional file 2: Table S2. Low to moderate assignment rates (28.6*–*56.4%) were found among *D. membranaceus *populations while considerably higher assignment rates (69.0*–*71.4%) occurred within* D. sinicus* populations. Among *D. membranaceus* populations, 57 out of 101 offspring (56.4%) of population A were successfully assigned to the most likely male parents, and 43 offspring (42.6%) were inferred as outcrossing, indicating that the mating pattern of population A is dominated by outcrossing. Within population B1, male parents of 100 *o*ut of 350 offspring (28.6%) were assigned, and 78 (22.3%) offspring were from outcrossing. Among the 240 offspring of population B2, 102 (42.5%) were successfully assigned to their male parents, including 56 selfing offspring and 46 outcrossing offspring, suggesting that selfing may be the main mating pattern within this population.

**Table 2:** Paternity assignments using COLONY

Species	Population	Total offspring	Assigned offspring	Assignment rate (%)	Selfing		Outcrossing	
					Amount	Percentage of total (%)	Amount	Percentage of total (%)
*D. membranaceus*	A	101	57	56.4	14	13.9	43	42.6
	B1	350	100	28.6	22	6.3	78	22.3
	B2	240	102	42.5	56	23.3	46	19.2
*D. sinicus*	C1	126	87	69.0	79	62.7	8	6.3
	C2	133	95	71.4	27	20.3	68	51.1

For *D. sinicus* populations, 87 offspring (69.0%) in population C1 and 95 offspring (71.4%) in population C2 were assigned to their male parents (Table 2). Of them, 62.7% offspring of population C1 were identified as selfing while only 20.3% offspring were identified as selfing and 51.1% as outcrossing in population C2, indicating that different mating patterns occurred within sporadically flowering populations of *D. sinicus*.

### Pollen dispersal distance and male reproductive contribution

We analyzed the male reproductive contribution of every flowering clump within five populations based on the result of paternity assignment (Table 3). Within population A, the maximum reproductive contribution was from clump A4 (42.1%), which produced 24 offspring. For population B1, both clumps FU4 and B2 produced 10 offspring, and had the maximum reproductive contribution (16.4%). Within population B2, clump C5 produced 53 offspring and had the maximum reproductive contribution (52.0%). For *D. sinicus* populations, the candidate male parent clump 13 in population C1 had a maximum reproductive contribution (73.6%) and produced 64 selfing offspring. Within population C2, clump 13B produced 66 offspring, and had reproductive contribution of 69.5%.

**Table 3:** Male reproductive contribution and pollen dispersal distance of *D. membranaceus* and *D. sinicus*

Species	Population	Candidate male parent	Female parent	Amount of offspring	Male reproductive contribution (%)	Pollen dispersal distance (m)
*D. membranaceus*	A	A0	A5, A6	4	7.0	60, 72
		A1	A4, A6	5	8.8	39, 61
		A2	A3, A4, A5, A6	15	26.3	16, 28, 39, 50
		A3	A2, A4, A5	5	8.8	16, 12, 23
		A4	A2, A3, A4, A5	24	42.1	28, 12, 0, 11
		A5	A4, A5, A6	4	7.0	11, 0, 11
		A6	–	–	–	–
		A7	–	–	–	–
	B1	FU1	B5, B6,	2	3.3	44, 34,
		FU2	B4, B7	3	4.9	27, 88
		FU3	B1, B2, B4, B5, B6	7	11.5	45, 43, 8, 11, 74
		FU4	B1, B2, B3, B4, B6, B7	10	16.4	49, 54, 52,72, 34, 37
		B1	B2, B4, B6	4	6.6	5, 37, 31
		B2	B1, B2	10	16.4	5, 0
		B3	B1, B4, B6, B7	5	8.2	6, 41, 30, 55
		B4	B1, B2, B3, B6, B7	7	11.5	37, 36, 41, 66, 90
		B5	B3, B5, B7	5	8.2	43, 0, 89
		B6	B3, B7	2	3.3	30, 25
		B7	B1, B2, B3, B4, B6, B7	6	9.8	56, 60, 55, 90, 25, 0
	B2	C1	C1, C4, C5	22	21.6	0, 23, 36
		C2	C1, C2, C3	15	14.7	12, 0, 13
		C3	C2, C3	2	2.0	13, 0
		C4	C2, C3, C4, C5	10	9.8	10, 8, 0, 16
		C5	C1, C3, C4, C5	53	52.0	36, 15, 26, 0
*D. sinicus*	C1	6	6, 13	23	26.4	0, 4378
		13	13	64	73.6	0
		5A	–	–	–	–
		5B	–	–	–	–
	C2	2	2	1	1.1	0
		3	2, 3, 13B	26	27.4	1113, 0, 1736
		13A	2	2	2.1	3338
		13B	3, 13A, 13B	66	69.5	1736, 1458, 0

Note: “-” indicated that offspring were not successfully assigned to the most likely male parents

**Fig. 2:**
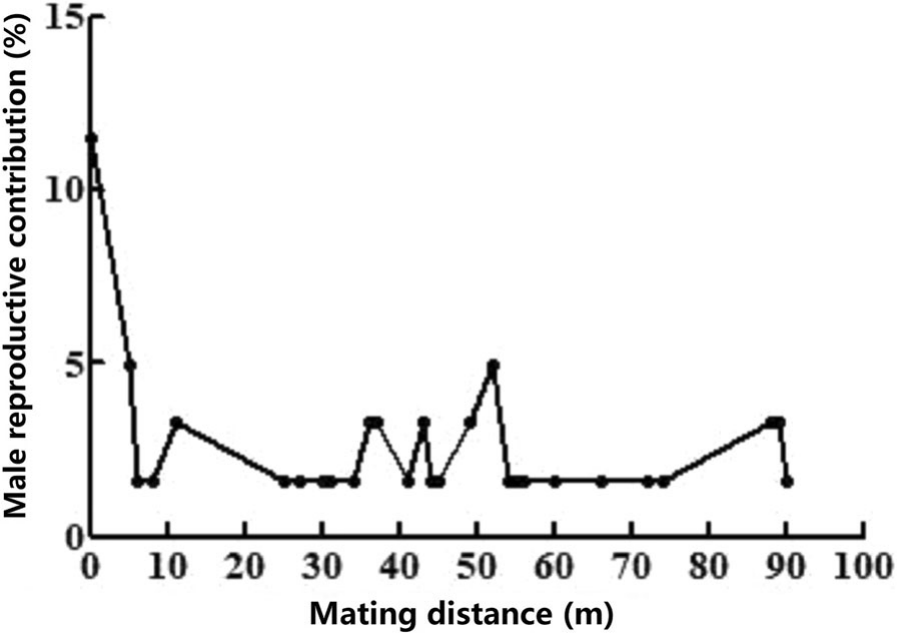
Relationships between male reproductive contribution and mating distance within flowering populations of *D. membranaceus*

According to the results of paternity analysis, we found that the maximum mating distance for sporadic and mass flowering populations of *D. membranaceus* were 72 m and 90 m, respectively. On the other hand, *D. sinicus* has a much longer mating distance (4378 m, Table 3). For sporadic flowering populations of *D. membranaceus* and *D. sinicus*, the mating distance was mainly concentrated on 0 m, indicating that most offspring were self-pollinated or geitonogamous. For mass flowering populations of *D. membranaceus*, the mating distance focused on a range of 0*–*50 m. Over 50 m, the amount of offspring reduced significantly with increasing geographical distance (Fig. 2).

Moreover, we did not find that candidate male parents preferred specific female parents to produce more offspring in *D. membranaceus* populations, while in sporadically flowering populations of *D. sinicus*, it appears that females tended to accept pollen from the closest males. For example, clumps 13A (female) and 13B (male) produced 43 outcrossing offspring, accounting for 63.2% of total outcrossing offspring in population C2 (Table 3, Additional file 2: Table S2).

## Discussion

### Mating system and variable mating patterns

Plant mating system is influenced by many factors, such as population density, floral synchronism, and post-pollination mechanisms [26]. In general, predominant selfing is common mating pattern within sporadically flowering populations of woody bamboos [18, 19, 21, 27], while predominant outcrossing is common in mass flowering populations [20]. In this study, a variable mating pattern of two tropical bamboo species was detected based on data from two consecutive flowering events over 2 years.

In a previous study, we inferred that both *D. membranaceu*s and* D. sinicus* had mixed mating systems with predominant outcrossing [16]. In the present study, we confirmed they were self-compatible. However their mating patterns changed depending on different flowering situations or pollination conditions. Predominant outcrossing was detected in both sporadic (population A in 2013) and mass (population B1 in 2013) flowering populations of *D. membranaceus*, while predominant selfing occurred in mass flowering population B2 in 2014. Similarly, sporadically flowering populations of *D. sinicus *demonstrated a mating pattern of predominant selfing in 2013 (population C1) and predominant outcrossing in 2015 (population C2).

The above observations might be associated with the flowering clump (or pollen) densities and foraging behavior of pollinator (e.g. honeybees) of bamboo populations [16, 22, 23]. Woody bamboos are generally considered to be wind-pollinated, and some studies have demonstrated that *Dendrocalamus* spp. are dichogamous and protogynous [16*–*18, 27]. A long flowering period (ca. one to 2 months) of *D. membranaceus* and *D. sinicus *allows the possibility of geitonogamous pollination within and among ramets [16, 21]. Mass flowering populations would provide greater pollen density than sporadically flowering populations. On the other hand, honeybees appeared to be good pollinators for *D. membranaceus *and *D. sinicus* [16]. Frequently clear and dry weather in 2013 is conducive for honeybees to pollinate. Therefore, populations A and B1 of *D. membranaceus* showed a mating pattern of predominant outcrossing. By comparison, commonly rainy weather during flowering period of *D. membranaceus* in 2014 hindered wind-pollination and honeybee pollination, thus decreased the outcrossing rate of population B2. As for *D. sinicus*, most seeds in 2015 (population C2) were from flowering clumps within less than 1800 m (Table 3), which was much shorter than interval distances of flowering clumps in 2013 (population C1). Short distances are beneficial to honeybees, and correspondingly increased the opportunity for outcrossing in population C2 more than in population C1 [28].

For breeding programs of woody bamboos, particularly programs based on seeds, the variable mating patterns will vastly increase the complexity of paternal genetic origin. This suggests that the first step in the breeding or selection of new bamboo varieties is to clarify genetic origin of the parents.

### Difference of paternity assignments rates between *D. membranaceus* and *D. sinicus*

Two sporadically flowering populations of *D. sinicus* (C1, C2) obtained relatively high paternity assignments rates (69.0–71.4%). Meanwhile, among three populations of *D. membranaceus*, the sporadically flowering population A also had much higher paternity assignments rates (56.4%) than mass flowering populations B1 (28.6%) and B2 (42.5%) (Table 2). Therefore, it was obvious that sporadically flowering populations (A, C1, C2) also displayed much higher paternity assignments rates than mass flowering populations (B1, B2). One of main reasons accounting for low assignment rates in mass flowering populations of *D. membranaceus* in the present study was integrity of the candidate male parents. Although we did not find other flowering clumps of *D. membranaceus* within the range of 200 m around populations A, B1 and B2 from 2012 to 2015, it was possible that honeybees frequently visited the fresh florets [16] and carried exogenous pollen into populations and resulted into a low assignment rates (28.6–56.4%). Compared with *D. membranaceus*, *D. sinicus* had usually rarer events of flowering and seeding. From 2013 to 2016, we did not observe other flowering clumps of *D. sinicus* within the range of ca. 10,000 m around populations C1 and C2. We thus probably collected all candidate male parents in populations C1 and C2, and obtained much higher assignment rates (69.0–71.4%).

Actually, low assignment rates in parentage analysis of natural populations in nature are common [29*–*31]. Similarly, within plant populations, high proportions of unassigned offspring are usually reported when there is large input from external pollen sources, particularly for wind-pollinated trees [32*–*35]. Therefore, the missing data of male parents and molecular markers with low resolution were two main reasons for low assignment rates [36–39]. Moreover, it was noteworthy that materials for paternity analysis in the present study came from seedlings of half-sib seeds. The seed germination rates of *D. membranaceus *and *D. sinicus *were only ca. 50*–*80% [40]. Thus, it would have a certain extent impact on the results of paternity analysis. The weak seeds or seedlings, particularly for selfing offspring, were excluded from paternity analysis, thus outcrossing rates would be artificially enlarged [19].

### Pollen dispersal distance

There was significant difference in pollen dispersal distance between *D. sinicus* and* D. membranaceus*. *Dendrocalamus sinicus* demonstrated a much greater pollen dispersal distance (4378 m) than *D. membranaceus* (90 m). Meanwhile, different from the viewpoint of Whitehead [41] that probability of mating success rate decreased with the increase of distance between female and male parents, optimum mating distances of *D. membranaceus* and *D. sinicus* mainly focused on a range of ca. 0*–*50 m and 0*–*1500 m respectively, with high male reproductive fitness.

Pollen dispersal distance is influenced by multiple factors, such as pollen amount, pollen viability, pollinator activity, weather and so on [16, 42]. Our results, in conjunction with previous studies, indicated that pollen amount and pollinator activity may be two of the most important factors for pollen dispersal distance and male reproductive fitness of* D. membranaceus *and *D. sinicus*.

The in vitro pollen of* D. membranaceus *and *D. sinicus* still had 38.1 and 4.8% germination ability respectively after being separated from flower branches for 3 hours (Yang et al. unpublished data), so that visiting flowers of honeybees could improve pollen dispersal distance and enhance pollination rates of geitonogamy and xenogamy [16]. However, bees generally had short flight ranges, so pollen movement in populations was biased towards nearby individuals [28]. Moreover, mass flowering or pollen limitation may also cause a short pollen dispersal to improve pollination [42*–*45].

In our previous studies, we found that *D. membranaceus* populations have denser flowering clumps and produced much more abundant pollen than those of *D. sinicus*, and pollination limitation in *D. sinicus* was more serious than that in *D. membranaceus* [16]. Therefore, these attributes may result in much shorter pollen dispersal in *D. membranaceus *populations. On the other hand, although *D. sinicus *demonstrated much farther pollen dispersal distance and optimum mating distance than *D. membranaceus*, it may be better reflected that interval distance between flowering clumps of *D. sinicus* were much farther away than those of *D. membranaceus*. As a whole, the total selfing rate of *D. sinicus* (40.9%) was much higher than that of *D. membranaceus *(13.3%, Table 2). When exogenous pollen is insufficient, self-pollination can improve the seed setting rate to ensure the survival of a species, that is, reproductive assurance effect [1, 4, 46].

## Conclusions

*Dendrocalamus membranaceus* and *D. sinicus* demonstrated mixed mating systems and their mating patterns changed depending on pollination conditions. *Dendrocalamus sinicus* had a much greater pollen dispersal distance than *D. membranaceus*. The mating distances of *D. membranaceus* and *D. sinicus* focused on a range of ca. 0–50 m and 0–1500 m respectively. These results also revealed the importance of pollinators, such as honeybees, in the mating systems of tropical woody bamboos.

## Materials and methods

### Study sites and seeds collection

**Fig. 3:**
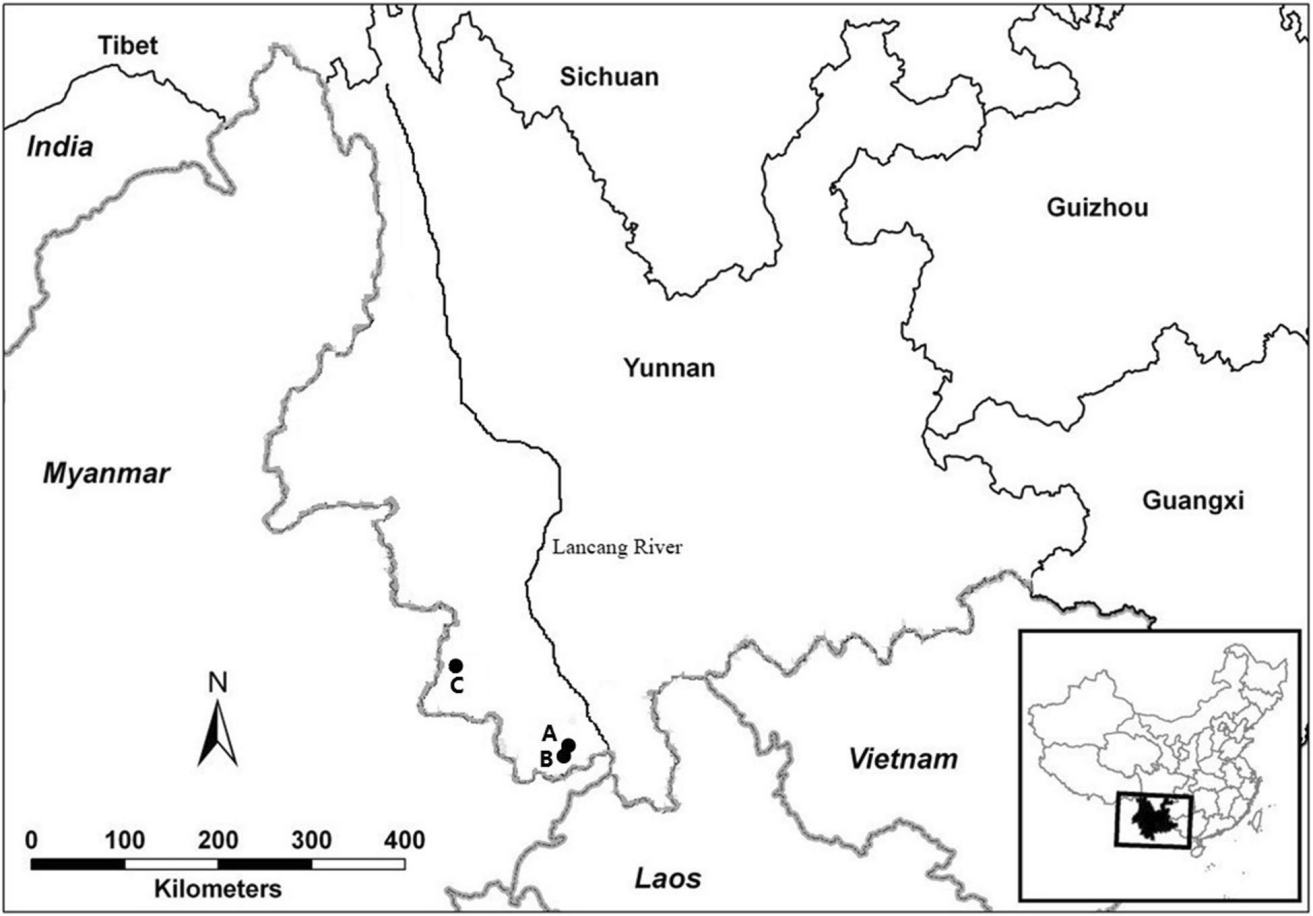
Location of study sites of *D. membranaceus* (sites A and B) and *D. sinicus* (site C) in Yunnan Province, China. Maps were drawn using the sofware ArcGIS version 10.2 (http://desktop.arcgis.com) and modified using Photoshop (Adobe Corporation, California, America)

The flowering populations of *Dendrocalamus membranaceus* studied in this paper occur in the Xiaomengyang National Nature Reserve (XNNR), Jinghong, Yunnan Province, China (Fig. 3). Mass and sporadic flowering of *D. membranaceus* occurred from 2011 to 2015 [23]. In 2013, we selected two fixed sites (i.e., A and B) for observation and seed collection (Table 4). Site A (sporadically flowering) was a bamboo-tree mixed forest (bamboo: tree = 9:1), and the area of quadrat was 10 × 100 m^2^. Eight flowering clumps were scattered among 55 total clumps (population A). The flowering clumps were not adjacent to each other and distances between flowering clumps were 16–72 m. Site B was in a pure *D. membranaceus* forest, and the area of quadrat was 40 × 100 m^2^. We found a small population in which all 12 clumps were flowering in 2013 and 2014, and treated it as a sample of mass flowering. No other flowering clumps were observed within a range of 200 m around this quadrat from 2012 to 2015. In July 2013 there were seven adjacent clumps (population B1) flowering in this quadrat, and the remaining five adjacent clumps (population B2) flowered in July 2014 (Table 4). The flowering periods of populations B1 and B2 did not overlapped.

**Table 4:** Study sites of paternity analyses for *D. membranaceus* and *D. sinicus*

Species	Study site	Longitude (E)	Latitude (N)	Elevation (m)	sampling date	Flowering type	Fruiting clumps	Offsprings	Candidate parent
*D. membranaceus*	A	100°52′32″	22°09′50″	810.0	2013.7	sporadic	6	101	8
	B1	100°52′27″	22°01′51″	756.8	2013.7	mass	7	350	11
	B2	100°52′27″	22°01′51″	756.8	2014.7	mass	5	240	5
*D. sinicus*	C1	99°31′36″	22°43′44″	1202.5	2013.6	sporadic	4	126	4
	C2	99°31′33″	22°43′48″	1295.0	2015.6	sporadic	4	133	4

The sporadically flowering clumps of *D. sinicus* were situated at the Wolong, Ximeng County, Yunnan Province (Fig. 3). In November 2012, four sporadically flowering clumps (population C1) were observed and seeds were collected in June 2013. At almost the same site, another four clumps (population C2) started flowering in January 2015.

The seeds of every population (A, B1, B2, C1 and C2) were collected from the flowering clumps in quadrats at XNNR or Wolong, and then marked with serial numbers (Tables 3 and 4). The voucher specimens of the flowering clumps were identified by Dr. Han-Qi Yang, and were deposited in the herbariums of the Research Institute of Resources Insects, Chinese Academy of Forestry (Kunming, China).

### Seed treatment

Following McClure [47], we regarded each clump as a potential genet and the culms within as ramets of a clone. Seeds collected from every flowering clump (Table 4) were sown in the greenhouse after treatments [40]. The leaves of seedlings were sampled and dried quickly with silica gel for DNA extractions. Each flowering genet in quadrats was identified as a candidate parent, and their foliage leaves were sampled for DNA extraction.

### Microsatellite analysis

Genomic DNA was extracted using the modified CTAB method [48] and dissolved in TE buffer (10 Mm Tris-HCL, pH 8.0, 1 Mm EDTA) to a final concentration of 30–60 ng/L [36]. We used 10 and 11 microsatellite loci (Additional file 3: Table S3) to genotype all the samples of *Dendrocalamus membranceus* and *D. sinicus* respectively with the software GeneMarker V. 2.2.0 (SoftGenetics, State College, PA, USA).

PCR amplification was performed according to Dong et al. [37] for *D. sinicus* and Dong and Yang [36] for *D. membranaceus*, respectively. PCR reactions were performed in 20 uL volumes, containing 1.0 uL DNA, 10 uL 2 × Taq PCR MasterMix, 8.6 uL ddH_2_O and 0.3 uL of each primer. Fluorescent-labelled primers were mixed with non-fluorescent primers. Cycling conditions included: 95 °C for 3 min followed by 34 cycles at 94 °C for 30 s, with an annealing temperature optimized for each primer pair for 40 s, 72 °C for 50 s, and a final extension step at 72 °C for 6 min. PCR products were visualized by silver staining on 6% polyacrylamide denaturing gels with a 20-bp molecular size standard ladder (Tiangen, Beijing, China).

### Mating system analysis

The program MLTR v3.2 [5] was used to assess the mating system from progeny arrays, which is based on maximum-likelihood. The following mating system parameters were estimated by the Newton-Raphson algorithm: multilocus outcrossing rate (t_m_), single-locus outcrossing rate (t_s_), and Biparental inbreeding (t_m_– t_s_). Standard deviation of these parameters was evaluated from 1000 bootstraps.

### Paternity analysis

We used the program COLONY 2.0 [25] to implement a paternity analysis for offspring of the sporadic and mass flowering populations. Error rate simulation was conducted several times until its value did not change. We performed three replicate runs and each run was performed with a different random number seed. The following parameters were adopted: monoecious species, allowing inbreeding, diploid, polygamy for males and females, full-likelihood method, medium length run, medium precision and no updating allele frequencies. We accepted the assignment for both paternity and maternity if results met one of the following criteria according to Harrison [49]: (1) all three runs were assigned to the same parent with a probability of 95% or more; (2) assigned to the same parent three times, and two of runs assigned above 95%; (3) two runs assigned the same parent, both above 95%, but another one runs failed to assign any candidate parent. Assignments were regarded as failed when the most likely male parents were in conflict among results of three replicate runs.

### Pollen dispersal and male reproductive success

Pollen dispersal was carried out for the sporadic and mass flowering populations based on paternity analysis. We deemed all female parent clumps (clumps with seed setting) as candidate male parent clumps in the studied populations, so the pollen dispersal distances were counted according to the geographical distances between female parent clump and inferred male parent clump based on results of paternity analysis. Male reproductive success (λ) was calculated by using the equation:

$$ {\uplambda}_{\mathrm{k}}={\mathrm{N}}_{\mathrm{k}}/\mathrm{N},\Sigma {\uplambda}_{\mathrm{k}}=1,\mathrm{for}\;\mathrm{K}=1,2,\dots, \mathrm{K}. $$

Where K is the number of candidate male parent, N_k_ is the number of offspring with known female parents and inferred male parent, and N is the total number of offspring [50, 51]. We also calculated the relationship between mating distance and male reproductive success.

## Supplementary information

**Additional file 1: Table S1.** Paternity analysis of *D. membranceus* using COLONY.

**Additional file 2: Table S2.** Paternity analysis of *D. sinicus* using COLONY.

**Additional file 3: Table S3.** The SSR primers were used for paternity analysis of *D. membranceus* and *D. sinicus*.

## Data Availability

All data generated or analyzed during this study are included in this published article (and its additional files).
