# Supplementary material for: Mixed mating system and variable mating patterns in tropical woody bamboos
Source: BMC Plant Biol. 2019 Oct 11;19:418. doi: 10.1186/s12870-019-2024-3 (PMC6787975; doi:10.1186/s12870-019-2024-3)
Supplement: Supplementary file 1 — Additional file 1: Table S1. Paternity analysis of D. membranceus using COLONY. [file 12870_2019_2024_MOESM1_ESM.doc]

**Additional Files**

**Table S1. Paternity analyses of *D. membranceus* using COLONY**

**Population A (Offspring in red indicated the selfing individual)**

| NO. | Offspring ID | Female parent | Assigned male parent |
| --- | --- | --- | --- |
| 1 | A2-1 | A2-MU | A3-MU |
| 2 | A2-4 | A2-MU | A4-MU |
| 3 | A2-5 | A2-MU | A4-MU |
| 4 | A2-7 | A2-MU | A4-MU |
| 5 | A3-5 | A3-MU | A4-MU |
| 6 | A3-6 | A3-MU | A4-MU |
| 7 | A3-7 | A3-MU | A2-MU |
| 8 | A3-10 | A3-MU | A4-MU |
| 9 | A3-13 | A3-MU | A4-MU |
| 10 | A3-15 | A3-MU | A4-MU |
| 11 | A3-17 | A3-MU | A4-MU |
| 12 | A4-1 | A4-MU | A1-MU |
| 13 | A4-2 | A4-MU | A4-MU |
| 14 | A4-3 | A4-MU | A4-MU |
| 15 | A4-6 | A4-MU | A1-MU |
| 16 | A4-7 | A4-MU | A1-MU |
| 17 | A4-8 | A4-MU | A4-MU |
| 18 | A4-10 | A4-MU | A1-MU |
| 19 | A4-11 | A4-MU | A4-MU |
| 20 | A4-12 | A4-MU | A2-MU |
| 21 | A4-14 | A4-MU | A4-MU |
| 22 | A4-15 | A4-MU | A4-MU |
| 23 | A4-16 | A4-MU | A4-MU |
| 24 | A4-17 | A4-MU | A5-MU |
| 25 | A4-18 | A4-MU | A5-MU |
| 26 | A4-19 | A4-MU | A5-MU |
| 27 | A4-20 | A4-MU | A4-MU |
| 28 | A4-21 | A4-MU | A4-MU |
| 29 | A4-22 | A4-MU | A4-MU |
| 30 | A4-24 | A4-MU | A3-MU |
| 31 | A4-25 | A4-MU | A4-MU |
| 32 | A4-26 | A4-MU | A2-MU |
| 33 | A4-28 | A4-MU | A4-MU |
| 34 | A4-29 | A4-MU | A3-MU |
| 35 | A4-30 | A4-MU | A2-MU |
| 36 | A4-31 | A4-MU | A4-MU |
| 37 | A5-2 | A5-MU | A2-MU |
| 38 | A5-7 | A5-MU | A3-MU |
| 39 | A5-8 | A5-MU | A0-MU |
| 40 | A5-13 | A5-MU | A2-MU |
| 41 | A5-14 | A5-MU | A2-MU |
| 42 | A5-16 | A5-MU | A2-MU |
| 43 | A5-20 | A5-MU | A2-MU |
| 44 | A5-21 | A5-MU | A2-MU |
| 45 | A5-23 | A5-MU | A0-MU |
| 46 | A5-26 | A5-MU | A2-MU |
| 47 | A5-27 | A5-MU | A2-MU |
| 48 | A5-29 | A5-MU | A5-MU |
| 49 | A5-30 | A5-MU | A4-MU |
| 50 | A5-31 | A5-MU | A3-MU |
| 51 | A5-32 | A5-MU | A2-MU |
| 52 | A5-33 | A5-MU | A0-MU |
| 53 | A5-34 | A5-MU | A2-MU |
| 54 | A6-1 | A6-MU | A2-MU |
| 55 | A6-4 | A6-MU | A5-MU |
| 56 | A6-5 | A6-MU | A1-MU |
| 57 | A6-6 | A6-MU | A0-MU |

**Population B1 (Offspring in red indicated the selfing individual)**

| No. | Offspring ID | Female parent | Assigned male parent |
| --- | --- | --- | --- |
| 1 | B1-8 | B1-MU | B2-MU |
| 2 | B1-13 | B1-MU | B-FU-2 |
| 3 | B1-15 | B1-MU | B-FU-4 |
| 4 | B1-16 | B1-MU | B-FU-2 |
| 5 | B1-19 | B1-MU | B-FU-2 |
| 6 | B1-21 | B1-MU | B1-MU |
| 7 | B1-22 | B1-MU | B-FU-2 |
| 8 | B1-26 | B1-MU | B6-MU |
| 9 | B1-28 | B1-MU | B-FU-3 |
| 10 | B1-29 | B1-MU | B2-MU |
| 11 | B1-33 | B1-MU | B-FU-2 |
| 12 | B1-41 | B1-MU | B7-MU |
| 13 | B1-44 | B1-MU | B6-MU |
| 14 | B1-48 | B1-MU | B-FU-3 |
| 15 | B1-49 | B1-MU | B7-MU |
| 16 | B2-1 | B2-MU | B5-MU |
| 17 | B2-3 | B2-MU | B-FU-2 |
| 18 | B2-4 | B2-MU | B2-MU |
| 19 | B2-6 | B2-MU | B5-MU |
| 20 | B2-12 | B2-MU | B5-MU |
| 21 | B2-19 | B2-MU | B5-MU |
| 22 | B2-21 | B2-MU | B2-MU |
| 23 | B2-23 | B2-MU | B2-MU |
| 24 | B2-24 | B2-MU | B2-MU |
| 25 | B2-29 | B2-MU | B-FU-4 |
| 26 | B2-31 | B2-MU | B6-MU |
| 27 | B2-33 | B2-MU | B-FU-3 |
| 28 | B2-34 | B2-MU | B2-MU |
| 29 | B2-35 | B2-MU | B6-MU |
| 30 | B2-37 | B2-MU | B6-MU |
| 31 | B2-38 | B2-MU | B1-MU |
| 32 | B2-39 | B2-MU | B2-MU |
| 33 | B2-40 | B2-MU | B2-MU |
| 34 | B2-45 | B2-MU | B2-MU |
| 35 | B2-47 | B2-MU | B6-MU |
| 36 | B2-48 | B2-MU | B2-MU |
| 37 | B2-49 | B2-MU | B2-MU |
| 38 | B2-50 | B2-MU | B6-MU |
| 39 | B3-2 | B3-MU | B6-MU |
| 40 | B3-4 | B3-MU | B-FU-4 |
| 41 | B3-6 | B3-MU | B6-MU |
| 42 | B3-10 | B3-MU | B5-MU |
| 43 | B3-12 | B3-MU | B6-MU |
| 44 | B3-13 | B3-MU | B6-MU |
| 45 | B3-17 | B3-MU | B-FU-4 |
| 46 | B3-21 | B3-MU | B1-MU |
| 47 | B3-41 | B3-MU | B-FU-1 |
| 48 | B3-45 | B3-MU | B3-MU |
| 49 | B3-50 | B3-MU | B6-MU |
| 50 | B4-3 | B4-MU | B7-MU |
| 51 | B4-4 | B4-MU | B5-MU |
| 52 | B4-12 | B4-MU | B3-MU |
| 53 | B4-14 | B4-MU | B5-MU |
| 54 | B4-15 | B4-MU | B7-MU |
| 55 | B4-20 | B4-MU | B5-MU |
| 56 | B4-22 | B4-MU | B-FU-3 |
| 57 | B4-24 | B4-MU | B5-MU |
| 58 | B4-25 | B4-MU | B-FU-3 |
| 59 | B4-26 | B4-MU | B-FU-3 |
| 60 | B4-27 | B4-MU | B-FU-3 |
| 61 | B4-29 | B4-MU | B5-MU |
| 62 | B4-34 | B4-MU | B-FU-3 |
| 63 | B4-35 | B4-MU | B5-MU |
| 64 | B4-37 | B4-MU | B-FU-3 |
| 65 | B4-38 | B4-MU | B-FU-1 |
| 66 | B4-41 | B4-MU | B-FU-3 |
| 67 | B4-42 | B4-MU | B5-MU |
| 68 | B4-45 | B4-MU | B5-MU |
| 69 | B4-47 | B4-MU | B-FU-3 |
| 70 | B4-49 | B4-MU | B-FU-3 |
| 71 | B5-3 | B5-MU | B-FU-3 |
| 72 | B5-8 | B5-MU | B-FU-3 |
| 73 | B5-20 | B5-MU | B-FU-3 |
| 74 | B5-22 | B5-MU | B5-MU |
| 75 | B5-27 | B5-MU | B5-MU |
| 76 | B5-34 | B5-MU | B1-MU |
| 77 | B5-38 | B5-MU | B-FU-1 |
| 78 | B5-45 | B5-MU | B-FU-3 |
| 79 | B6-1 | B6-MU | B5-MU |
| 80 | B6-8 | B6-MU | B2-MU |
| 81 | B6-11 | B6-MU | B3-MU |
| 82 | B6-17 | B6-MU | B-FU-2 |
| 83 | B6-26 | B6-MU | B1-MU |
| 84 | B6-34 | B6-MU | B1-MU |
| 85 | B6-43 | B6-MU | B6-MU |
| 86 | B7-4 | B7-MU | B-FU-4 |
| 87 | B7-20 | B7-MU | B1-MU |
| 88 | B7-24 | B7-MU | B-FU-1 |
| 89 | B7-25 | B7-MU | B7-MU |
| 90 | B7-26 | B7-MU | B7-MU |
| 91 | B7-28 | B7-MU | B-FU-3 |
| 92 | B7-30 | B7-MU | B7-MU |
| 93 | B7-31 | B7-MU | B7-MU |
| 94 | B7-33 | B7-MU | B5-MU |
| 95 | B7-34 | B7-MU | B1-MU |
| 96 | B7-35 | B7-MU | B3-MU |
| 97 | B7-36 | B7-MU | B7-MU |
| 98 | B7-38 | B7-MU | B7-MU |
| 99 | B7-45 | B7-MU | B7-MU |
| 100 | B7-46 | B7-MU | B-FU-2 |

**Population B2 (Offspring in red indicated the selfing individual)**

| No. | Offspring ID | Female parent | Assigned male parent |
| --- | --- | --- | --- |
| 1 | C1-3 | C1-MU | C5-MU |
| 2 | C1-8 | C1-MU | C1-MU |
| 3 | C1-9 | C1-MU | C1-MU |
| 4 | C1-13 | C1-MU | C1-MU |
| 5 | C1-19 | C1-MU | C1-MU |
| 6 | C1-20 | C1-MU | C1-MU |
| 7 | C1-21 | C1-MU | C5-MU |
| 8 | C1-23 | C1-MU | C1-MU |
| 9 | C1-26 | C1-MU | C1-MU |
| 10 | C1-27 | C1-MU | C1-MU |
| 11 | C1-28 | C1-MU | C1-MU |
| 12 | C1-30 | C1-MU | C1-MU |
| 13 | C1-32 | C1-MU | C5-MU |
| 14 | C1-34 | C1-MU | C1-MU |
| 15 | C1-35 | C1-MU | C2-MU |
| 16 | C1-36 | C1-MU | C1-MU |
| 17 | C1-40 | C1-MU | C1-MU |
| 18 | C1-42 | C1-MU | C1-MU |
| 19 | C1-43 | C1-MU | C1-MU |
| 20 | C1-44 | C1-MU | C1-MU |
| 21 | C1-47 | C1-MU | C1-MU |
| 22 | C2-1 | C2-MU | C2-MU |
| 23 | C2-2 | C2-MU | C4-MU |
| 24 | C2-3 | C2-MU | C4-MU |
| 25 | C2-7 | C2-MU | C2-MU |
| 26 | C2-8 | C2-MU | C2-MU |
| 27 | C2-9 | C2-MU | C2-MU |
| 28 | C2-10 | C2-MU | C4-MU |
| 29 | C2-11 | C2-MU | C2-MU |
| 30 | C2-12 | C2-MU | C2-MU |
| 31 | C2-13 | C2-MU | C3-MU |
| 32 | C2-15 | C2-MU | C2-MU |
| 33 | C2-19 | C2-MU | C4-MU |
| 34 | C2-21 | C2-MU | C2-MU |
| 35 | C2-26 | C2-MU | C2-MU |
| 36 | C2-30 | C2-MU | C2-MU |
| 37 | C2-32 | C2-MU | C2-MU |
| 38 | C2-33 | C2-MU | C2-MU |
| 39 | C2-34 | C2-MU | C4-MU |
| 40 | C2-36 | C2-MU | C2-MU |
| 41 | C3-15 | C3-MU | C4-MU |
| 42 | C3-16 | C3-MU | C4-MU |
| 43 | C3-20 | C3-MU | C5-MU |
| 44 | C3-21 | C3-MU | C5-MU |
| 45 | C3-29 | C3-MU | C5-MU |
| 46 | C3-31 | C3-MU | C4-MU |
| 47 | C3-34 | C3-MU | C2-MU |
| 48 | C3-48 | C3-MU | C3-MU |
| 49 | C4-2 | C4-MU | C5-MU |
| 50 | C4-3 | C4-MU | C4-MU |
| 51 | C4-5 | C4-MU | C5-MU |
| 52 | C4-6 | C4-MU | C1-MU |
| 53 | C4-8 | C4-MU | C5-MU |
| 54 | C4-12 | C4-MU | C5-MU |
| 55 | C4-15 | C4-MU | C5-MU |
| 56 | C4-17 | C4-MU | C5-MU |
| 57 | C4-18 | C4-MU | C5-MU |
| 58 | C4-19 | C4-MU | C5-MU |
| 59 | C4-21 | C4-MU | C5-MU |
| 60 | C4-22 | C4-MU | C5-MU |
| 61 | C4-24 | C4-MU | C5-MU |
| 62 | C4-27 | C4-MU | C5-MU |
| 63 | C4-29 | C4-MU | C5-MU |
| 64 | C4-30 | C4-MU | C5-MU |
| 65 | C4-32 | C4-MU | C5-MU |
| 66 | C4-34 | C4-MU | C5-MU |
| 67 | C4-35 | C4-MU | C5-MU |
| 68 | C4-36 | C4-MU | C5-MU |
| 69 | C4-42 | C4-MU | C5-MU |
| 70 | C4-44 | C4-MU | C5-MU |
| 71 | C4-45 | C4-MU | C5-MU |
| 72 | C4-48 | C4-MU | C5-MU |
| 73 | C4-49 | C4-MU | C5-MU |
| 74 | C5-3 | C5-MU | C5-MU |
| 75 | C5-4 | C5-MU | C5-MU |
| 76 | C5-7 | C5-MU | C5-MU |
| 77 | C5-9 | C5-MU | C4-MU |
| 78 | C5-11 | C5-MU | C5-MU |
| 79 | C5-13 | C5-MU | C5-MU |
| 80 | C5-14 | C5-MU | C5-MU |
| 81 | C5-16 | C5-MU | C5-MU |
| 82 | C5-17 | C5-MU | C1-MU |
| 83 | C5-18 | C5-MU | C5-MU |
| 84 | C5-19 | C5-MU | C5-MU |
| 85 | C5-20 | C5-MU | C5-MU |
| 86 | C5-22 | C5-MU | C5-MU |
| 87 | C5-23 | C5-MU | C5-MU |
| 88 | C5-24 | C5-MU | C5-MU |
| 89 | C5-26 | C5-MU | C5-MU |
| 90 | C5-27 | C5-MU | C5-MU |
| 91 | C5-28 | C5-MU | C5-MU |
| 92 | C5-31 | C5-MU | C5-MU |
| 93 | C5-34 | C5-MU | C1-MU |
| 94 | C5-35 | C5-MU | C1-MU |
| 95 | C5-36 | C5-MU | C5-MU |
| 96 | C5-38 | C5-MU | C5-MU |
| 97 | C5-41 | C5-MU | C5-MU |
| 98 | C5-43 | C5-MU | C5-MU |
| 99 | C5-44 | C5-MU | C1-MU |
| 100 | C5-45 | C5-MU | C5-MU |
| 101 | C5-46 | C5-MU | C5-MU |
| 102 | C5-48 | C5-MU | C5-MU |
