# Supplementary material for: Mixed mating system and variable mating patterns in tropical woody bamboos
Source: BMC Plant Biol. 2019 Oct 11;19:418. doi: 10.1186/s12870-019-2024-3 (PMC6787975; doi:10.1186/s12870-019-2024-3)
Supplement: Supplementary file 2 — Additional file 2: Table S2. Paternity analysis of D. sinicus using COLONY. [file 12870_2019_2024_MOESM2_ESM.doc]

**Additional Files**

**Table S2 Paternity analyses of *D. sinicus* using COLONY**

**Population C1 (Offspring in red indicated the selfing individual)**

| No. | Offspring ID | Female parent | Assigned male parent |
| --- | --- | --- | --- |
| 1 | 13-1 | 13-MU | 13-FU |
| 2 | 13-2 | 13-MU | 13-FU |
| 3 | 13-4 | 13-MU | 13-FU |
| 4 | 13-5 | 13-MU | 13-FU |
| 5 | 13-7 | 13-MU | 13-FU |
| 6 | 13-10 | 13-MU | 13-FU |
| 7 | 13-11 | 13-MU | 13-FU |
| 8 | 13-12 | 13-MU | 13-FU |
| 9 | 13-13 | 13-MU | 13-FU |
| 10 | 13-14 | 13-MU | 13-FU |
| 11 | 13-16 | 13-MU | 13-FU |
| 12 | 13-19 | 13-MU | 13-FU |
| 13 | 13-20 | 13-MU | 13-FU |
| 14 | 13-21 | 13-MU | 13-FU |
| 15 | 13-22 | 13-MU | 13-FU |
| 16 | 13-23 | 13-MU | 13-FU |
| 17 | 13-24 | 13-MU | 13-FU |
| 18 | 13-25 | 13-MU | 13-FU |
| 19 | 13-26 | 13-MU | 13-FU |
| 20 | 13-27 | 13-MU | 13-FU |
| 21 | 13-28 | 13-MU | 13-FU |
| 22 | 13-30 | 13-MU | 13-FU |
| 23 | 13-31 | 13-MU | 13-FU |
| 24 | 13-32 | 13-MU | 13-FU |
| 25 | 13-33 | 13-MU | 13-FU |
| 26 | 13-34 | 13-MU | 13-FU |
| 27 | 13-35 | 13-MU | 13-FU |
| 28 | 13-36 | 13-MU | 13-FU |
| 29 | 13-37 | 13-MU | 13-FU |
| 30 | 13-38 | 13-MU | 13-FU |
| 31 | 13-39 | 13-MU | 13-FU |
| 32 | 13-40 | 13-MU | 13-FU |
| 33 | 13-41 | 13-MU | 13-FU |
| 34 | 13-42 | 13-MU | 13-FU |
| 35 | 13-43 | 13-MU | 13-FU |
| 36 | 13-44 | 13-MU | 13-FU |
| 37 | 13-46 | 13-MU | 13-FU |
| 38 | 13-47 | 13-MU | 13-FU |
| 39 | 13-48 | 13-MU | 13-FU |
| 40 | 13-49 | 13-MU | 13-FU |
| 41 | 13-52 | 13-MU | 13-FU |
| 42 | 13-53 | 13-MU | 13-FU |
| 43 | 13-54 | 13-MU | 13-FU |
| 44 | 13-55 | 13-MU | 13-FU |
| 45 | 13-57 | 13-MU | 13-FU |
| 46 | 13-58 | 13-MU | 13-FU |
| 47 | 13-59 | 13-MU | 13-FU |
| 48 | 13-60 | 13-MU | 13-FU |
| 49 | 13-61 | 13-MU | 13-FU |
| 50 | 13-63 | 13-MU | 13-FU |
| 51 | 13-64 | 13-MU | 13-FU |
| 52 | 13-65 | 13-MU | 13-FU |
| 53 | 13-66 | 13-MU | 13-FU |
| 54 | 13-67 | 13-MU | 13-FU |
| 55 | 13-68 | 13-MU | 13-FU |
| 56 | 13-70 | 13-MU | 13-FU |
| 57 | 13-71 | 13-MU | 13-FU |
| 58 | 13-72 | 13-MU | 13-FU |
| 59 | 13-73 | 13-MU | 13-FU |
| 60 | 13-74 | 13-MU | 13-FU |
| 61 | 13-76 | 13-MU | 13-FU |
| 62 | 13-77 | 13-MU | 13-FU |
| 63 | 13-78 | 13-MU | 13-FU |
| 64 | 13-79 | 13-MU | 13-FU |
| 65 | 6-1 | 6-MU | 13-FU |
| 66 | 6-2 | 6-MU | 6-FU |
| 67 | 6-3 | 6-MU | 13-FU |
| 68 | 6-4 | 6-MU | 6-FU |
| 69 | 6-6 | 6-MU | 6-FU |
| 70 | 6-7 | 6-MU | 13-FU |
| 71 | 6-9 | 6-MU | 13-FU |
| 72 | 6-10 | 6-MU | 6-FU |
| 73 | 6-12 | 6-MU | 6-FU |
| 74 | 6-13 | 6-MU | 6-FU |
| 75 | 6-14 | 6-MU | 6-FU |
| 76 | 6-15 | 6-MU | 13-FU |
| 77 | 6-16 | 6-MU | 13-FU |
| 78 | 6-17 | 6-MU | 6-FU |
| 79 | 6-18 | 6-MU | 6-FU |
| 80 | 6-20 | 6-MU | 13-FU |
| 81 | 6-21 | 6-MU | 6-FU |
| 82 | 6-22 | 6-MU | 6-FU |
| 83 | 6-23 | 6-MU | 6-FU |
| 84 | 6-25 | 6-MU | 6-FU |
| 85 | 6-26 | 6-MU | 6-FU |
| 86 | 6-27 | 6-MU | 13-FU |
| 87 | 6-28 | 6-MU | 6-FU |

**Population C2 (Offspring in red indicated the selfing individual)**

| No. | Offspring ID | Female parent | Assigned male parent |
| --- | --- | --- | --- |
| 1 | 2-1 | 2-MU | 2-FU |
| 2 | 2-2 | 2-MU | 3-FU |
| 3 | 2-3 | 2-MU | 3-FU |
| 4 | 2-8 | 2-MU | 13A-FU |
| 5 | 2-10 | 2-MU | 13A-FU |
| 6 | 3-2 | 3-MU | 3-FU |
| 7 | 3-7 | 3-MU | 3-FU |
| 8 | 3-10 | 3-MU | 3-FU |
| 9 | 3-12 | 3-MU | 13B-FU |
| 10 | 13A-2 | 13A-MU | 13B-FU |
| 11 | 13A-3 | 13A-MU | 13B-FU |
| 12 | 13A-4 | 13A-MU | 13B-FU |
| 13 | 13A-5 | 13A-MU | 13B-FU |
| 14 | 13A-6 | 13A-MU | 13B-FU |
| 15 | 13A-7 | 13A-MU | 13B-FU |
| 16 | 13A-8 | 13A-MU | 13B-FU |
| 17 | 13A-9 | 13A-MU | 13B-FU |
| 18 | 13A-10 | 13A-MU | 13B-FU |
| 19 | 13A-11 | 13A-MU | 13B-FU |
| 20 | 13A-12 | 13A-MU | 13B-FU |
| 21 | 13A-14 | 13A-MU | 13B-FU |
| 22 | 13A-15 | 13A-MU | 13B-FU |
| 23 | 13A-16 | 13A-MU | 13B-FU |
| 24 | 13A-17 | 13A-MU | 13B-FU |
| 25 | 13A-18 | 13A-MU | 13B-FU |
| 26 | 13A-19 | 13A-MU | 13B-FU |
| 27 | 13A-20 | 13A-MU | 13B-FU |
| 28 | 13A-21 | 13A-MU | 13B-FU |
| 29 | 13A-22 | 13A-MU | 13B-FU |
| 30 | 13A-23 | 13A-MU | 13B-FU |
| 31 | 13A-24 | 13A-MU | 13B-FU |
| 32 | 13A-25 | 13A-MU | 13B-FU |
| 33 | 13A-26 | 13A-MU | 13B-FU |
| 34 | 13A-27 | 13A-MU | 13B-FU |
| 35 | 13A-29 | 13A-MU | 13B-FU |
| 36 | 13A-30 | 13A-MU | 13B-FU |
| 37 | 13A-31 | 13A-MU | 13B-FU |
| 38 | 13A-32 | 13A-MU | 13B-FU |
| 39 | 13A-33 | 13A-MU | 13B-FU |
| 40 | 13A-34 | 13A-MU | 13B-FU |
| 41 | 13A-35 | 13A-MU | 13B-FU |
| 42 | 13A-36 | 13A-MU | 13B-FU |
| 43 | 13A-39 | 13A-MU | 13B-FU |
| 44 | 13A-40 | 13A-MU | 13B-FU |
| 45 | 13A-41 | 13A-MU | 13B-FU |
| 46 | 13A-43 | 13A-MU | 13B-FU |
| 47 | 13A-44 | 13A-MU | 13B-FU |
| 48 | 13A-45 | 13A-MU | 13B-FU |
| 49 | 13A-46 | 13A-MU | 13B-FU |
| 50 | 13A-47 | 13A-MU | 13B-FU |
| 51 | 13A-48 | 13A-MU | 13B-FU |
| 52 | 13A-49 | 13A-MU | 13B-FU |
| 53 | 13B-2 | 13B-MU | 13B-FU |
| 54 | 13B-3 | 13B-MU | 13B-FU |
| 55 | 13B-4 | 13B-MU | 13B-FU |
| 56 | 13B-6 | 13B-MU | 13B-FU |
| 57 | 13B-7 | 13B-MU | 13B-FU |
| 58 | 13B-8 | 13B-MU | 13B-FU |
| 59 | 13B-9 | 13B-MU | 13B-FU |
| 60 | 13B-10 | 13B-MU | 13B-FU |
| 61 | 13B-11 | 13B-MU | 13B-FU |
| 62 | 13B-12 | 13B-MU | 13B-FU |
| 63 | 13B-13 | 13B-MU | 13B-FU |
| 64 | 13B-14 | 13B-MU | 13B-FU |
| 65 | 13B-15 | 13B-MU | 13B-FU |
| 66 | 13B-16 | 13B-MU | 13B-FU |
| 67 | 13B-17 | 13B-MU | 13B-FU |
| 68 | 13B-18 | 13B-MU | 13B-FU |
| 69 | 13B-19 | 13B-MU | 13B-FU |
| 70 | 13B-20 | 13B-MU | 13B-FU |
| 71 | 13B-21 | 13B-MU | 13B-FU |
| 72 | 13B-22 | 13B-MU | 13B-FU |
| 73 | 13B-24 | 13B-MU | 13B-FU |
| 74 | 13B-25 | 13B-MU | 13B-FU |
| 75 | 13B-28 | 13B-MU | 3-FU |
| 76 | 13B-29 | 13B-MU | 3-FU |
| 77 | 13B-30 | 13B-MU | 13B-FU |
| 78 | 13B-31 | 13B-MU | 3-FU |
| 79 | 13B-32 | 13B-MU | 3-FU |
| 80 | 13B-34 | 13B-MU | 3-FU |
| 81 | 13B-35 | 13B-MU | 3-FU |
| 82 | 13B-36 | 13B-MU | 3-FU |
| 83 | 13B-37 | 13B-MU | 3-FU |
| 84 | 13B-38 | 13B-MU | 3-FU |
| 85 | 13B-39 | 13B-MU | 3-FU |
| 86 | 13B-40 | 13B-MU | 3-FU |
| 87 | 13B-41 | 13B-MU | 3-FU |
| 88 | 13B-42 | 13B-MU | 3-FU |
| 89 | 13B-43 | 13B-MU | 3-FU |
| 90 | 13B-44 | 13B-MU | 3-FU |
| 91 | 13B-45 | 13B-MU | 3-FU |
| 92 | 13B-46 | 13B-MU | 3-FU |
| 93 | 13B-47 | 13B-MU | 3-FU |
| 94 | 13B-48 | 13B-MU | 3-FU |
| 95 | 13B-49 | 13B-MU | 3-FU |
