# Supplementary material for: Mixed mating system and variable mating patterns in tropical woody bamboos
Source: BMC Plant Biol. 2019 Oct 11;19:418. doi: 10.1186/s12870-019-2024-3 (PMC6787975; doi:10.1186/s12870-019-2024-3)
Supplement: Supplementary file 3 — Additional file 3: Table S3. The SSR primers were used for paternity analysis of D. membranceus and D. sinicus. [file 12870_2019_2024_MOESM3_ESM.doc]

**Additional Files**

***Table S3. The SSR primers were used for paternity analyses of D. membranceus and D. sinicus***

| Species | Locus | Primer sequences (5’–3’) | Repeat motif | GenBank accession no. | Literature |
| --- | --- | --- | --- | --- | --- |
| *D.membranaceus* | Mun-33 | F: CCTTTTCACCGTTCGATATGT  R: AAGTTGGTTGGAGACGGATAG | (TC)10 | KF553643 | Dong and Yang, 2014 |
|  | Mun-45 | F: GCGCTTACACCAACACCTCAT  R: CTTGCGTGGAACCGAGAAC | (TC)10 | KF553644 | Dong and Yang, 2014 |
|  | Mun-59 | F: CCCTAAACAACCTGCAACACT  R: CGTTTTATCGGGCTATTGGAG | (CA)10 | KF553646 | Dong and Yang, 2014 |
|  | Mun-63 | F: CACCCCATTCGTACC  R: ATTCTTTGTTAGGCTCTTCT | (AG)9...(AG)5 | KF553648 | Dong and Yang, 2014 |
|  | Mun-66 | F: TAACCACAAGCACGCAAT  R: TAAGATGGGCATGAGAGG | (AG)9 | KF553649 | Dong and Yang, 2014 |
|  | Mun-72 | F: ATCCAACGAGAAACGAAG  R: TGGTACGCATCAGCTAAG | (CT)8 | KF553651 | Dong and Yang, 2014 |
|  | Mun-74 | F: GTGGCATATTGGAAGTGAG  R: CGACGATAAAGTAGGTTGG | (AC)9 | KF553652 | Dong and Yang, 2014 |
|  | Mun-80 | F: TGCAAGAAAACATCACGC  R: AGTCTATGCCTCCGGTTATTC | (CA)6 | KF553654 | Dong and Yang, 2014 |
|  | Mun-96 | F: TATTTTTCGTCCTCTAGCTG  R: TTAGGTGCTTGCAGAATGATGG | (TG)8 | KF553656 | Dong and Yang, 2014 |
|  | Den-075 | F: ATTATGTCGGCAAAAAAGCAGA  R: CGGTCAATGTGAAGTGTCT | (TG)7 | JQ366050 | Dong *et al*., 2012 |
| *D. sinicus* | Mun-45 | F:GCGCTTACACCAACACCTCAT  R:CTTGCGTGGAACCGAGAAC | （TC) 10 | KF553644 | Dong and Yang, 2014 |
|  | Mun-63 | F:CACCCCATTCGTACC  R:ATTCTTTGTTAGGCTCTTCT | (AG) 9…(AG) 5 | KF553648 | Dong and Yang, 2014 |
|  | Den-006 | F: GTCAGGAGGCACAACAAAAT  R: GACCTCTGCTTTCGGATAA | (TC) 8 | JQ366033 | Dong *et al*., 2012 |
|  | Den-033 | F: AGGGAGGCTTGGTGCTTAGA  R: CTCACTGTAGTCGCATCAAA | (TA) 7 (TG) 8 | JQ366042 | Dong *et al*., 2012 |
|  | Den-036 | F: ATCTAGGTGGTATGAACAAT  R: CGTATGTATTTGTGTATCGG | (TA) 6 (TG) 11 | JQ366044 | Dong *et al*., 2012 |
|  | Den-075 | F:ATTATGTCGGCAAAAAAGCAGA  R:CGGTCAATGTGAAGTGTCT | (TG) 7 | JQ366050 | Dong *et al*., 2012 |
|  | DLTGMS-472 | F: GAGGCATTAAGCACAGAATTA  R: GCGAAAGCTTAAACTCTATCC | (GA) 11 | Pr032402678 | Bhandawat *et al*., 2016 |
|  | DLTGMS-730 | F: CAGGAGGAGGAAGGAACC  R: GTGTATCTCTTGATGCTGTCC | (CT) 13 | Pr032405545 | Bhandawat *et al*., 2016 |
|  | DLUGMS-17 | F:CGGTTGGCCTTCTATGAGAG  R:CCATCGATGATAGCACAGGA | (TC) 24 | JK013403.1 | Bhandawat *et al*., 2016 |
|  | BOM-02 | F:TTGGAGGAGACTGAATGA  R:CCTTTGCGAAAATACACG | (GT) 10 | EE661488 | Dong *et al*., 2011 |
|  | 4373 | F: CAAGACAGTGGCTTCCTCCTC  R: ACCGAACAAACGGACAGTGC | (TTC) 14 | NM175737.3 | Gao *et al*., 2011 |
